# Addendum to brachytherapy dose‐volume histogram commissioning with multiple planning systems

**DOI:** 10.1120/jacmp.v17i3.6105

**Published:** 2016-05-08

**Authors:** Michael S. Gossman

**Affiliations:** ^1^ Regulation Directive Medical Physics Department of Radiation Oncology KY USA; ^2^ Exponent, Inc., Division of Biomedical Engineering Philadelphia PA USA; ^3^ Tri‐State Regional Cancer Center Department of Radiation Oncology Ashland KY 41101 USA; ^4^ Regulation Directive Medical Physics Flatwoods KY USA

**Keywords:** brachytherapy, dose, DVH, histogram, volume, JACMP

## Abstract

The process for validating dose‐volume histogram data in brachytherapy software is presented as a supplement to a previously published article. Included is the DVH accuracy evaluation of the Best NOMOS treatment planning system called “Best TPS VohvmePlan” As done previously in other software, a rectangular cuboid was contoured in the treatment planning system. A single radioactive ^125^I source was positioned coplanar and concentric with one end. Calculations were performed to estimate dose deposition in partial volumes of the cuboid structure, using the brachytherapy dosimetry formalism defined in AAPM Task Group 43. Hand‐calculated, dose‐volume results were compared to TPS‐generated, point‐source‐approximated dose‐volume histogram data to establish acceptance. The required QA for commissioning was satisfied for the DVH as conducted previously for other software, using the criterion that the DVH $\%{\text{Vol}}_{\text{TPS}}$ “actual variance” calculations should differ by no more than 5% at any specific radial distance with respect to $\%{\text{Vol}}_{\text{TG}‐43}$, and the “average variance” DVH $\%{\text{Vol}}_{\text{TPS}}$ calculations should differ by no more than 2% over all radial distances with respect to $\%{\text{Vol}}_{\text{TG}‐43}$. The average disagreement observed between hand calculations and treatment planning system DVH was less than 0.5% on average for this treatment planning system and less than 1.1% maximally for 1≤r≤5 cm.

PACS number(s): 87.10.+e, 87.55.‐x, 87.53.Jw, 07.05.Tp

## I. INTRODUCTION

The dose‐volume histogram (DVH) is regarded by radiation oncologists as essential for their consideration of the appropriateness of a brachytherapy treatment plan.^(1)^ For this reason, DVH metrics reported by a treatment planning systems (TPS) should be inspected at the time of commissioning, as well as on a routine basis, to insure calculation accuracy. Although the AAPM Task Group 53 (TG‐53) report mentions DVH quality assurance (QA) should be conducted, there are no traditional tolerances or detailed recommendations provided for such testing.^(2)^ Merely a need to do it is discussed in literature.^3^, ^4^, ^5^, ^6^, ^7^, ^8^ In a first‐ever attempt to strategize an accurate assessment of dose‐volume statistics for brachytherapy software, we published an article last year which proposed the basic tolerances for concluded acceptance.^(9)^ The required QA for commissioning and routine testing examines whether the DVH percentage volume “actual variance” calculations differs by no more than 5% at any specific radial distance with respect to hand‐calculated percentage volume, and if the “average variance” DVH percentage volume calculations differ by no more than 2% over all radial distances with respect to the hand‐calculated percentage volume.^(9)^ In that former research, quality assurance of DVH statistics were quantified and validated for various low dose‐rate (LDR) and high dose‐rate (HDR) software. Included were brachytherapy treatment planning systems: Varian Medical Systems, Inc. (Palo Alto, CA) VariSeed and BrachyVision; Philips Healthcare (Amsterdam, Netherlands) Pinnacle^3^; MIM Software, Inc. (Cleveland, OH) Symphony; and the Nucletron (Elekta, Stockholm, Sweden) Oncentra. We now provide treatment planning system DVH quality assurance evaluation of the Best NOMOS (DBA: Best Medical International, Inc., Pittsburgh, PA), Best TPS VolumePlan, as the final missing currently marketed brachytherapy software.

## II. MATERIALS AND METHODS

As conducted in the original article written by Gossman et al.,^(9)^ a Model 6711 (GE Healthcare–Oncura, Inc., Arlington Heights, IL) sealed source was chosen for use in LDR DVH verification with the Best TPS VolumePlan software. Standards of formalism provided in the AAPM Task Group 43 (TG‐43) report and updates TG‐43U1 and TG‐43U1S1 were instituted to yield an accurate calculation of absorbed dose in a permanent implant using the point‐source approximation.^10^, ^11^, ^12^ A narrow rectangular cuboid having dimensions $0.5\;\text{cm}\times 0.5\;\text{cm}\times 5.0\;\text{cm}\;(1.25\;{\text{cm}}^{3})$ was delineated on various planning images. The volume began with a rectangular contour drawn on 2 slices immediately superior and 3 slices inferior to the slice containing the origin for a net contoured width of 0.5 cm in 6 slices. This resulted in a DVH calculated volume of $1.242\;{\text{cm}}^{3}$. This was a mere 0.6% from the known volume. A single $I^{125}$ brachytherapy source was assigned to the left center edge of the cuboid, with the source axis perpendicular to the long axis of the cuboid. The Model 6711 source strength 1.01 U was arbitrarily assigned to deliver a permanent implant dose of 20 Gy at the reference position. Best TPS VolumePlan Release 5.0‐DASM was then enabled to calculate the percentage isodose values around the source, which project laterally outward throughout the volume of the cuboid. The process results in a unique isodose line for each measureable length along the cuboid. Each incremental length of the cuboid was correlated with the volume irradiated at the isodose line shown. Statistical data comparatively were pulled from the DVH curve generated by the TPS. Partial dose‐volume data, hand‐calculated using TG‐43 formalism, were then directly compared to DVH software results to ascertain acceptance.

## III. RESULTS AND DISCUSSION

It was geometrically known that, with a contoured cuboid structure length of 5.0 cm, it then followed that for every 1.0 cm away from the source, the dose delivered at that point incrementally envelopes approximately 20% of the structure of the cuboid. The result enables tabulation of $\%{\text{Vol}}_{\text{TG}‐43}$ along with the absorbed dose at that distance, as denoted by $\%{\text{Dose}}_{\text{TG}‐43}$. As shown previously in the original article written by Gossman et al.,^(9)^ correction factors for isotropic source falloff were then applied to hand calculations for a beam with a flattening filter. Volumetric correction factors were found to be 1.045, 1.021, 1.014, 1.010, and 1.008 for radial distances 1 cm, 2 cm, 3 cm, 4 cm, and 5 cm, respectively. With the inclusion of Best TPS VolumePlan by Best NOMOS in all TPS software evaluated for $I^{125}$, Table 1 provides the parameters and calculations involved for the determination of dose to five distances from the Model 6711 ^125^I source, with cumulative plots for all results in Fig. 1. Nucletron Oncentra is not tabulated here, since it was used in the previous publication for an HDR source only.

**Table 1:** Cumulative brachytherapy TPS DVH statistical ranges for $I^{125}$ vs. TG‐43 results.

*Source Model*	*%Dose*	*VeriSeed*	*TPS %Vol Difference BrachyVision*	${Pinnacle}^{3}$	*Symphony*	*VolumePlan*
6711.0	100.0	0.3	0.8	0.1	0.3	0.6
	20.5	0.2	0.9	−0.1	−0.1	1.0
	7.1	0.3	2.0	0.5	0.8	0.9
	3.1	0.5	3.6	0.2	4.9	0.5
	1.5	0.6	−4.3	0.1	−2.2	−1.1
	Average	0.4	0.6	0.2	0.7	0.4
	Range	0.2‐0.6	−4.3−3.6	−0.1−0.5	−2.2−4.9	−1.1−1.0

**Figure 1:**
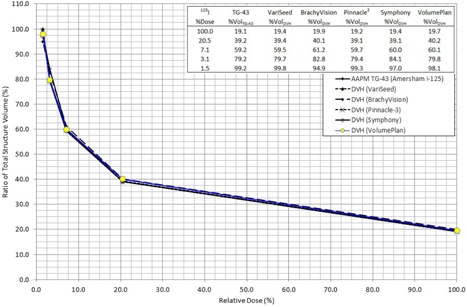
Cumulative brachytherapy TPS DVH results for $I^{125}$ vs. TG‐43 predicted doses.

The average results for all systems at all distances and for all sources were within 0.5%. No TPS calculated differences exceeding ±5% at specific points of analysis. The maximum difference computed by comparison to TG‐43 hand‐calculated predicted values were only −1.1%. Care should be taken to ensure the highest resolution and accuracy when contouring in order to minimize errors that affect $\%{\text{Vol}}_{\text{TG}‐43}$ directly.

## IV. CONCLUSIONS

Using identical methodology as incorporated previously, the average disagreement observed between hand calculations and the TPS DVH was less than ±1% for the TPSs studied.^(9)^ The variation at each distance (1 cm≤r≤5 cm) was within 5% for all data, including that of the Best NOMOS Release 5.0 DASM of the Best TPS VolumePlan software. It is advocated as a practice standard for brachytherapy TPS DVH quality assurance, that if the DVH $\%{\text{Vol}}_{\text{TPS}}$ “actual variance” calculations differ by more than 5% at any radial distance with respect to $\%{\text{Vol}}_{\text{TG}‐43}$, or if the DVH $\%{\text{Vol}}_{\text{TPS}}$ “average variance” calculations differ by more than 2% over all radial distances with respect to $\%{\text{Vol}}_{\text{TG}‐43}$, that the disparity should be investigated. Here, the Best TPS VolumePlan software also passed these performance criteria using TG‐43 formalism.

## ACKNOWLEDGMENTS

This research was conducted with appreciation to President Krishnan Suthanthiran, ME, and R&D Director Vineet Gupta, PhD, at Best NOMOS (Pittsburgh, PA), who provided the software for evaluation on request.

## COPYRIGHT

This work is licensed under a Creative Commons Attribution 4.0 International License.
